# Impact of pharmacists as immunizers on influenza vaccination coverage in the community-setting in Nova Scotia, Canada: 2013-2015

**DOI:** 10.1186/s40545-016-0084-4

**Published:** 2016-10-19

**Authors:** Jennifer E. Isenor, Jessica L. Killen, Beverly A. Billard, Shelly A. McNeil, Donna MacDougall, Beth A. Halperin, Kathryn L. Slayter, Susan K. Bowles

**Affiliations:** 1 College of Pharmacy, Dalhousie University, 5968 College Street, PO Box 15000, Halifax, Nova Scotia B3H 4R2 Canada; 2 Canadian Center for Vaccinology, IWK Health Centre and Nova Scotia Health Authority, Dalhousie University, 5850/5980 University Ave, Halifax, Nova Scotia B3K 6R8 Canada; 3 Faculty of Medicine, Dalhousie University, 1459 Oxford St, Halifax, Nova Scotia B3H 4R2 Canada; 4 Nova Scotia Department of Health and Wellness, PO Box 488, Halifax, Nova Scotia B3J 2R8 Canada; 5 School of Nursing, St. Francis Xavier University, PO Box 5000, Antigonish, Nova Scotia B2G 2W5 Canada; 6 School of Nursing, Dalhousie University, 5869 University Avenue, PO Box 15000, Halifax, Nova Scotia B3H 4R2 Canada; 7 IWK Health Centre, 5850/5980 University Avenue, Halifax, Nova Scotia B3K 6R8 Canada; 8 Department of Pharmacy, 1796 Summer St, Nova Scotia Health Authority- Central Zone, Halifax, Nova Scotia B3H 3A6 Canada

**Keywords:** Pharmacist, Immunization, Influenza vaccination, Pharmaceutical services, Vaccine coverage, Canada

## Abstract

**Background:**

Annual immunization is the most effective way to prevent influenza and its associated complications. However, optimal immunization rates are not being met in Nova Scotia, Canada. Additional providers, such as pharmacists, may improve access and convenience to receive vaccines. Pharmacists began immunizing patients 5 years of age and older within the publicly funded universal influenza vaccination program during the 2013-2014 influenza season. The objective of this study was to evaluate influenza immunization coverage rates before and after pharmacists in Nova Scotia gained authority to immunize as part of the publicly funded universal influenza vaccination program.

**Methods:**

Influenza immunization data was obtained from the Department of Health and Wellness from 2010 to 2015. Data included billing data from physicians and pharmacists, and local public health data. Vaccination coverage was calculated as proportion of vaccinations received in comparison to the total population.

**Results:**

Prior to pharmacists immunizing, overall vaccination coverage for Nova Scotia residents 6 months of age and older was 35.8 % in 2012-2013, increasing to 41.8 % coverage in 2013-2014 the year pharmacists began immunizing. A decrease of 1.9 to 39.9 % was observed in 2014-2015. In patients 65 years of age and older living in the community, coverage has increased from 61.8 % in 2012-2013 to 71.6 % in 2013-2014, and again to 73.3 % in 2014-2015 with the addition of pharmacists immunizing. Prior to pharmacists immunizing the highest coverage noted for this portion of the population was 61.8 %.

**Conclusions:**

The addition of pharmacists as immunizers within a publicly funded universal influenza vaccination program was found to increase overall vaccination coverage in the first year and to maintain higher coverage rates in the second year than those observed before pharmacists began immunizing. Increases in coverage in both years were observed in the elderly. Future research will be required to determine the ongoing impact of the addition of pharmacists as immunizers and other strategies to improve vaccination coverage.

## Background

Receiving the seasonal influenza vaccine each year is the most effective way for an individual to protect themselves from influenza [1, 2]. Globally, influenza rates are estimated between 5 to 10 % for adults and 20 to 30 % among children. Worldwide, this translates to an approximate three to five million cases of severe influenza per year, with an estimated 250,000 to 500,000 deaths attributed to influenza [3]. Influenza is of greatest concern to those who are at high risk of complications, such as the elderly, pregnant women, or those with co-morbidities [1, 4].

Annual influenza immunization is the most effective way to reduce the risk of influenza-related complications. As such, several jurisdictions have identified goals for vaccine coverage rates [5–7], for example, the Office of Disease Prevention and Health Promotion in the United States has set annual influenza vaccine targets at 80 % for noninstitutionalized individuals aged 18 to 64 years, and 90 % for anyone aged 18 years and older who is institutionalized and for health care personnel [6]. In Canada, the National Advisory Committee on Immunization (NACI) has set vaccine coverage goals of 80 % for people 65 years of age or older, those younger than 65 years of age with high risk comorbidities, and health care workers; 95 % for residents of long term care; and 100 % coverage for vaccinators [7]. There are many challenges to reaching these goals, including accessibility and convenience of obtaining vaccinations. In Canada, pharmacists are viewed as highly trusted health care providers with the potential to decrease these barriers and improve vaccination coverage [8, 9]. In 2013, legislation was introduced in Nova Scotia (NS), Canada that allowed pharmacists, with appropriate certification, the authority to immunize adults and children 5 years of age and older [8]. Currently, pharmacists are able to administer the influenza vaccine in 9 of 10 provinces in Canada [10]. In 2013-2014, pharmacists in NS were able to provide influenza vaccines as part of the publicly funded universal vaccination program, which had been implemented in 2010 [11]. Although data from the United States, where pharmacists have been providing immunization services since 1995, demonstrate increased uptake of influenza immunization with the addition of pharmacist immunizers, little is known about the impact of pharmacist immunizers in Canada [12–15]. In British Columbia, pharmacy vaccination clinics were introduced and immunization rates in older people increased [13]. Our evaluation of the first year that pharmacists provided immunization services within the publicly funded universal influenza program showed an increase in overall vaccination rates, despite a decrease in vaccinations administered by physicians and public health [12].

This study expands on the previous NS data and compares NS influenza immunization rates prior to the addition of pharmacists as immunizers, with the 2 years following introduction of immunization services by pharmacists within the publicly funded universal influenza vaccination program [12].

### Aim of the study

The aim of this study was to compare estimated influenza vaccination coverage in Nova Scotia, Canada in the 3 years prior to the expansion of pharmacist scope of practice to include administration of vaccinations with the 2 years in which pharmacists were permitted to administer influenza vaccines.

## Methods

Census data and aggregate immunization data, which included physician and pharmacist billings and local public health data, were obtained from the Nova Scotia Department of Health and Wellness (DHW). Data was provided as number of vaccinations per district in the health authority by each type of care provider—physicians, pharmacists and public health/other (which included occupational health employees and home care nurses), and by year (end-August one year, to end-August the following year). For the purposes of comparison 2010-2011, 2011-2012 and 2012-2013 were defined as the pre-pharmacist involvement influenza seasons; 2013-2014 and 2014-2015 were considered the post-pharmacist involvement seasons. Vaccination coverage by provider was defined as the percentage of Nova Scotia residents vaccinated by each provider type, in relation to the entire population of Nova Scotia. Although pharmacists can only provide immunizations to those 5 years of age and older, data for those 6 months and older was used when comparing coverage between providers due to missing age related data necessary for alternate comparisons.

### Data analysis

Estimated vaccination coverage and 95 % confidence intervals for influenza immunization were constructed for each year using the number of influenza immunizations administered as the numerator, and the census data most proximal to the year of interest as the denominator (2010-2011and 2011-2012 used 2006 census data; 2012-2013 and 2013-2014 used 2011 census data and 2014-2015 used 2013 census data). Coverage was calculated using total populations and estimates grouped by age and sex, and compared between vaccination provider groups. Comparisons between the proportions of the immunized populations each year were made using Chi-Square with Bonferronni correction for multiple comparisons. All data analysis was completed using EXCEL XLSTAT version 2015.

## Results

In 2013-2014, 430 pharmacists in NS were licensed to immunize, and in 2014-2015, 293 additional pharmacists were licensed to vaccinate, bringing the total to 723.

**Fig. 1:**
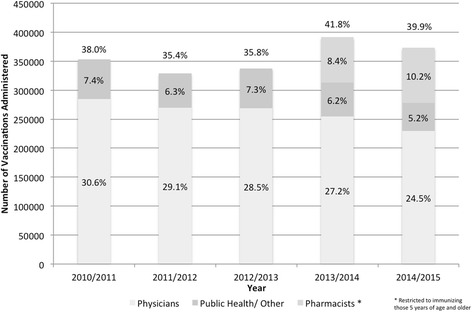
Number of vaccinations administered and vaccination coverage by year and provider type represented as coverage of total population (%) for Nova Scotia residents 6 months of age or older

In 2014-2015, overall vaccination coverage for all Nova Scotia residents 6 months of age or older decreased by 1.9 % (95 % CI, 1.76–2.04) from 41.8 % (95 % CI, 41.68–41.88) in 2013-2014 to 39.9 % (95 % CI, 39.78–39.98) (Fig. 1). The highest vaccination coverage achieved before pharmacists began immunizing was 38 % (95 % CI, 37.93–38.13) in 2010-2011. In 2011-2012 and 2012-2013 vaccination coverage was between 35 % and 36 %. Comparison of immunization rates by gender did not show any differences between years, with a ratio of approximately 55 % of vaccine recipients being female and 45 % being male.

**Fig. 2:**
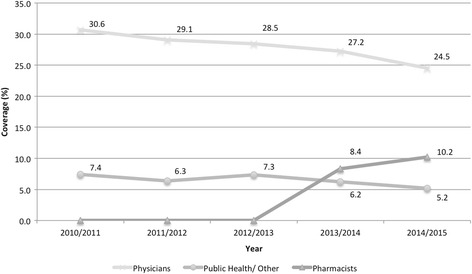
Trends in influenza vaccination coverage by provider type for patients 6 months of age or older

The vaccination coverage provided by pharmacists increased between 2013-2014 and 2014-2015 from 8.4 to 10.2 % (Fig. 2). The vaccination coverage provided by physicians to patients 6 months of age or older decreased from 27.2 % in 2013-2014 to 24.5 % in 2014-2015 (Fig. 2). Likewise, the coverage provided by public health also decreased from 6.2 to 5.2 % between 2013-2014 and 2014-2015 (Fig. 2).

**Fig. 3:**
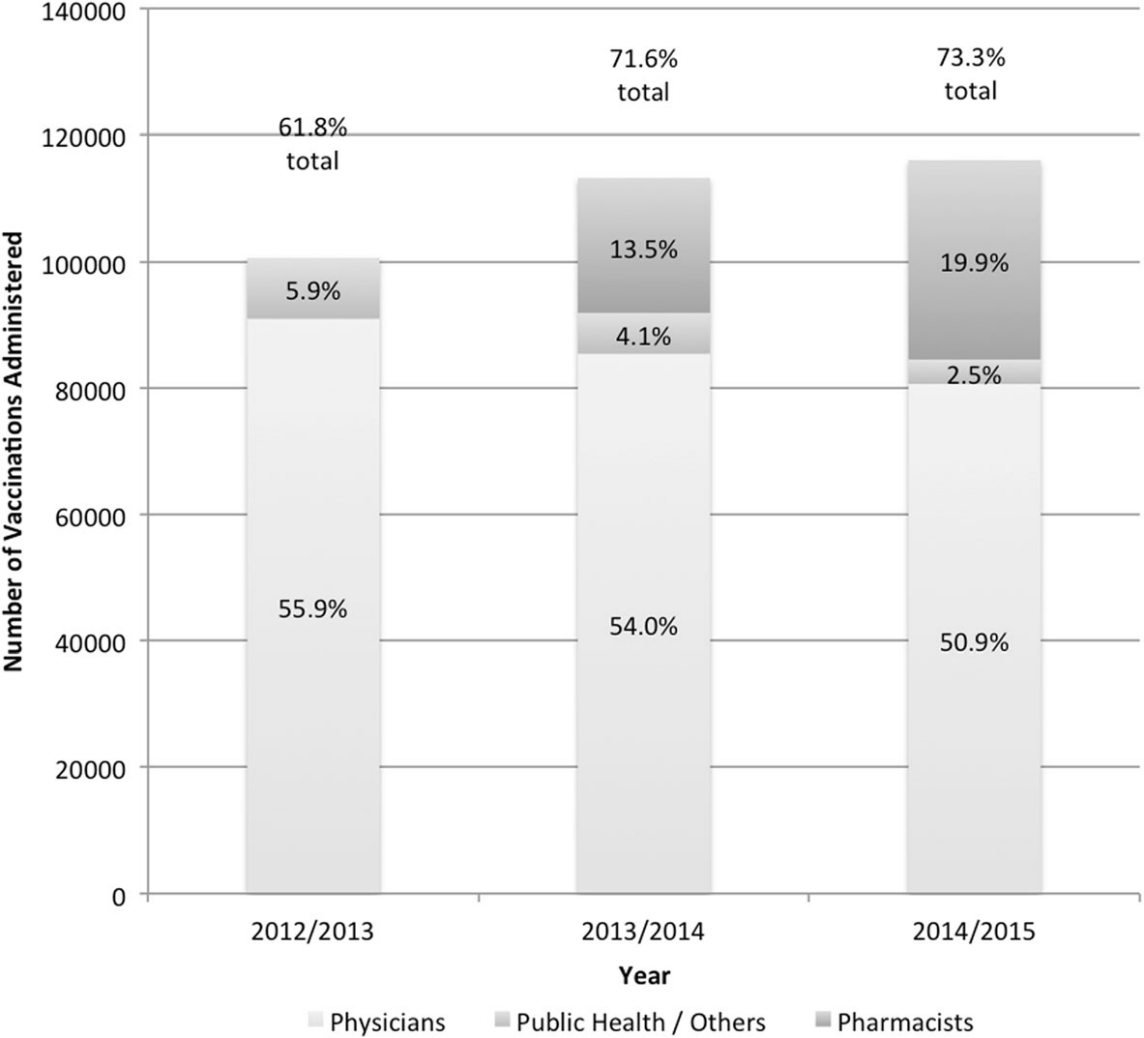
Vaccination coverage by provider group and total population coverage for Nova Scotia residents 65 years of age and older, excluding those in long term care

For individuals 65 years of age and older, overall vaccination coverage increased by 9.8 % (95 % CI, 9.5–10.2) to 71.6 % (95 % CI, 71.4–71.8) in the first year that pharmacists began immunizing, and increased by another 1.7 % (95 % CI, 1.4–2.0) in the second year, resulting in overall coverage of 73.3 % (95 % CI, 73.1–73.5) in 2014-2015 (Fig. 3). Physicians remained the primary providers of immunizations in this age group (Fig. 3). Prior to pharmacists immunizing, overall coverage rates in this population were 61.8 % (95 % CI, 61.5–62.0) in 2012-2013. The data were unavailable for years prior to 2012. A decrease in immunization coverage was noted for children 6 to 59 months from 45.4 % coverage in 2013-2014 to 40.6 % in 2014-2015, an age group that pharmacists are not permitted to immunize.

The vaccination coverage provided by pharmacists to the age groups 18–23 years and 24–64 years remained relatively unchanged from the 2013-2014 influenza vaccination season to the 2014-2015 season. There was a decrease in the coverage provided to 5–17 year olds compared to 2013-2014 data (12.5 to 9.9 %), whereas there was an increase in the coverage provided to those 65 years and older (27.4 to 33.1 %).

## Discussion

Our data demonstrate that the increase observed in influenza immunization rates immediately following implementation of pharmacist-provided immunization services within the universal influenza program was sustained in the second year of the program. Although a 1.9 % (95 % CI, 1.76–2.04) reduction in overall rates was observed between the first and second year of pharmacists in the immunizer role, immunization rates remained higher than those observed prior to the introduction of pharmacist immunizers in NS. Of note, immunization coverage declined in 2014-2015 in those 6 to 59 months of age, whom pharmacists are not permitted to immunize, and therefore contributed to the overall decline in rates, independent of pharmacists’ ability to immunize. Furthermore, immunization rates continued to increase among those 65 years of age and older, an age group that pharmacists are permitted to immunize.

Limitations of this study include potential errors related to secondary use of health claims aggregate data, such as errors in age-related coding, gender coding or improper reporting and billing. Missing age related data in 2012-2013 prevented complete comparisons for the age range that pharmacists can provide immunizations (5 years of age and older). In assessing vaccinations for patients less than 9 years of age, it is not possible to assess the proportion of patients who received two doses of influenza vaccine as recommended in the first year vaccinated against influenza, an important consideration given the lack of efficacy of a single dose in this group [11]. However, over-reporting should be consistent in all years evaluated and by all providers. In addition, the number of children between 5 and 9 years of age who were immunized by pharmacists was small; therefore the overall impact on results would be expected to be minimal. Other potential confounders that may have resulted in an increase or decrease in coverage over the years studied include the 2009 H1N1 pandemic and potential residual effects on coverage in 2010-2011, and a perceived vaccine shortage in 2013-2014 related to a distribution delay [16, 17]. Decreased public confidence due to widespread media coverage regarding poor vaccine effectiveness secondary to a mismatch with the predominant circulating strain in 2014-2015 is an additional confounder, which may have resulted in decreased vaccine uptake that year [11].

Higher rates for vaccination coverage noted in 2010-2011 may have been associated with the first year of the universally funded influenza vaccination program, or the residual impact of the pandemic H1N1 seen in 2009-2010 [16]. There is no apparent reason why coverage decreased from 2011 to 2013. The highest vaccine coverage in the years studied was observed in the 2 years that included pharmacist involvement in the program with the first year being the highest, possibly in part due to additional advertisements from pharmacies and increased media attention and news reports on the addition of pharmacists to the influenza vaccination campaign in the province. The slight decrease in vaccination coverage observed in 2014-2015 could be related to the reduced vaccine effectiveness observed in the 2013-2014 season during which the vaccine match to the dominant circulating H3N2 strain was poor [18].

These data lend further support to the benefit of pharmacist involvement in influenza vaccination programs in Canada or countries with similar health systems. Data from 2013-2014, the first year that pharmacists provided immunization services in NS, demonstrated an overall increase in vaccination coverage with the addition of pharmacists as immunizers and not merely a shift, although there is some, of vaccinations from other providers to pharmacists [12]. This is consistent with data from the United States that showed the benefit of pharmacists in increasing overall influenza immunization rates, which showed that those over 18 years of age were more likely to become vaccinated when the vaccine was provided at their community pharmacy [14].

With respect to NS data, a steady decline in the number of vaccinations provided by physicians each year was observed, leading to the question of whether immunization services previously provided by physicians are now being substituted by pharmacists. Arguing against this is that the rate of decline began prior to pharmacists providing influenza vaccination (Fig. 2). The reasons for the decrease in the proportion of vaccinations administered by physicians are likely complex, involving both practitioner and patient factors. Possible explanations include increased responsibility for physicians, leading to time constraints and longer wait times for each patient, and shorter patient-physician interactions resulting in less time to provide immunizations [19, 20]. In NS and other parts of Canada, perceived shortages and concerns around distribution of family physicians may be a barrier to patient access, which could directly lead to fewer vaccinations administered by physicians [21, 22].

Vaccination coverage by public health, while varying from year to year, has remained relatively steady between 5 and 8 % annually. The decrease in vaccinations administered by public health in 2013-2014 and 2014-2015 was expected because fewer public health clinics were offered as a conscious effort to allow public health practitioners to focus on other activities since pharmacists were authorized to also provide influenza vaccination.

By the end of the second year of pharmacist-provided immunization, there was an increase in coverage from 71.6 to 73.3 % in those individuals greater than 65 years of age, approaching the national goal of 80 % coverage for community dwelling seniors [2]. The overall increase in coverage in this population was not observed across all immunization provider groups. While pharmacists and public health saw an increase in the number of elderly being vaccinated, the number of older adults immunized by physicians continued to decline. Between 2013-2014 and 2014-2015, the demographics of the population being vaccinated by pharmacists changed, with an increase in the proportion being administered to the elderly, and a decreasing proportion being administered to all other age groups. This could indicate that people 65 years of age and older are an important demographic for pharmacists to engage or that this age group is most readily accessed by pharmacists. In general, older adults visit their pharmacy more frequently than younger people because they have more chronic conditions, providing more opportunity for pharmacists to make vaccination recommendations to this population [23]. As elderly are also at higher risk of complications, pharmacists may make greater effort to ensure vaccination in this population than in younger people presenting to their pharmacy. It may also relate to the confidence that older individuals have in pharmacists such that they are more willing to be vaccinated by a pharmacist than younger individuals. Our findings are consistent with both US and Canadian data that also observed patients 65 years of age and older were more likely to become immunized with the addition of pharmacists as immunizers [13, 14].

With regards to gender, there have been no significant changes in coverage noted with the addition of pharmacists as immunizers, however each year there is a significant difference between more females than males receiving their influenza vaccination. This could be associated with the fact that women see their physician more regularly than men, or visit their pharmacy more often than men to pick up prescriptions, allowing physicians and pharmacists to make a recommendation for vaccinations [24]. This difference was also observed in 2001 by Grabenstein et al., noting that 60 % of patients receiving immunizations from 21 pharmacies in the US were female [25].

## Conclusions

The addition of pharmacists as immunizers has resulted in an increase in influenza vaccine coverage rates in Nova Scotia, Canada. The impact appears to be greatest among adults who are 65 years of age or older and was sustained over the first 2 years in which pharmacists vaccinated. Future studies should address the impact of the addition of pharmacists over a longer time period and explore the potential for targeted interventions by pharmacists to increase vaccine coverage among other at-risk populations.

## References

[1] National Advisory Committee on Immunization (NACI). Statement on seasonal influenza vaccine for 2014-2015. http://www.phac-aspc.gc.ca/naci-ccni/flu-grippe-eng.php (2014). Accessed 05 Aug 2015.

[2] Public Health Agency of Canada. Vaccine coverage amongst adult Canadians: Results from the 2012 adult National Immunization Coverage (aNIC) survey. http://www.phac-aspc.gc.ca/im/nics-enva/vcac-cvac-eng.php. (2014). Accessed 06 Jun 2015.

[3] World Health Organization. Influenza (seasonal) fact sheet #211. http://www.who.int/mediacentre/factsheets/fs211/en/ (2014). Accessed 21 Feb 2016.

[4] Bowles S, Strang R, Jovaisas B (2014). Infectious diseases: influenza. Compendium of therapeutic choices.

[5] NHS England Department of Health. The national flu immunisation programme 2015/16. https://www.gov.uk/government/uploads/system/uploads/attachment_data/file/418428/Annual_flu_letter_24_03_15__FINALv3_para9.pdf (2015). Accessed 16 May 2016.

[6] Office of Disease Prevention and Health Promotion. Immunization and infectious diseases. https://www.healthypeople.gov/2020/topics-objectives/topic/immunization-and-infectious-diseases/objectives (2016). Accessed 16 May 2016.

[7] Public Health Agency of Canada (2007). Final report of outcomes from the national consensus conference for vaccine-preventable diseases in Canada. CCDR.

[8] Canadian Pharmacists Association. Environmental scan: pharmacy practice legislation and policy changes across Canada. blueprintforpharmacy.ca/docs/kt- tools/environmental-scan---pharmacy-practice-legislation-and-policy-changes-may-2014.pdf (2014). Access 06 Jun 2015.

[9] Kimmel SR, Burns IT, Wolfe RM, Zimmerman RK (2007). Addressing immunization barriers, benefits, and risks. J Fam Pract.

[10] Canadian Pharmacists Association. Pharmacists’ expanded scope of practice. http://www.pharmacists.ca/pharmacy-in-canada/scope-of-practice-canada/ (2016). Accessed 21 Feb 2016.

[11] Public Health Agency of Canada. Statement on seasonal influenza vaccine for 2015-2016. http://www.phac-aspc.gc.ca/naci-ccni/flu-2015-grippe-eng.php (2016). Accessed 12 May 2016.

[12] Isenor JE, Alia TA, Killen JL, Billard BA, Halperin BA, Slayter KL, McNeil SA, MacDougall D, Bowles SK (2016). Impact of pharmacists as immunizers on influenza vaccination coverage in Nova Scotia, Canada. Hum Vaccin Immunother.

[13] Marra F, Kaczorowski J, Gastonguay L, Lynd LD, Kendall P (2014). Pharmacy-based immunization in rural communities strategy (PhICS): A community cluster-randomized trial. Can Pharm J.

[14] Steyer TE, Ragucci KR, Pearson WS, Mainous AG (2004). The role of pharmacists in the delivery of influenza vaccinations. Vaccine.

[15] Grabenstein JD, Guess HA, Hartzema AG, Konrad TR (2001). Effect of vaccination by community pharmacists among adult prescription recipients. Med Care.

[16] Centers for Disease Control and Prevention.The 2009 H1N1 pandemic: summary highlights. http://www.cdc.gov/h1n1flu/cdcresponse.htm (2010). Accessed 16 Jan 2016.

[17] CBC News. Flu shots across Nova Scotia in high demand. http://www.cbc.ca/news/canada/nova-scotia/flu-shots-across-nova-scotia-in-high-demand-1.2490598 (2014). Accessed 16 Jan 2016.

[18] Centers for Disease Control and Prevention. Morbidity and mortality weekly report (MMWR): Influenza activity- United States, 2014-15 season and composition of the 2015-16 influenza vaccine. http://www.cdc.gov/mmwr/preview/mmwrhtml/mm6421a5.htm (2015). Accessed 10 Jan 2016.

[19] Braddock CH, Snyder L (2005). The doctor will see you shortly. The ethical significance of time for the patient-physician relationship. J Gen Intern Med.

[20] Dugdale DC, Epstein R, Pantilat SZ (1999). Time and patient-physician relationship. J Gen Intern Med.

[21] CBC News. Nova Scotia doctor shortage needs action, ex-medical dean says. http://www.cbc.ca/news/canada/nova-scotia/nova-scotia-doctor-shortage-needs-action-ex-medical-dean-says-1.3137431 (2015). Accessed 16 Jan 2016.

[22] DesRoches J. Myth: Canada needs more doctors. http://www.cfhi-fcass.ca/SearchResultsNews/12-05-29/80fe1ee3-444d-4114-b9ee-d9da20439293.aspx (2012). Accessed 26 May 2016.

[23] Canadian Association of Chain Drug Stores. 9000 points of care: improving access to affordable health care. http://9000pointsofcare.ca (2014). Accessed 26 Aug 2015.

[24] Centers for Disease Control and Prevention. New study profiles women’s use of health care. http://www.cdc.gov/nchs/pressroom/01news/newstudy.htm (2010). Accessed 06 Jan 2016.

[25] Grabenstein JD, Guess HA, Hartzema AG (2001). People vaccinated by pharmacists: Descriptive epidemiology. J Am Pharm Assoc.

